# Molecular and Cellular Mechanisms of Aldosterone Producing Adenoma Development

**DOI:** 10.3389/fendo.2015.00095

**Published:** 2015-06-11

**Authors:** Sheerazed Boulkroun, Fabio Luiz Fernandes-Rosa, Maria-Christina Zennaro

**Affiliations:** ^1^UMRS_970, Paris Cardiovascular Research Center, Institut National de la Santé et de la Recherche Médicale (INSERM), Paris, France; ^2^University Paris Descartes, Sorbonne Paris Cité, Paris, France; ^3^Assistance Publique-Hôpitaux de Paris, Hôpital Européen Georges Pompidou, Service de Génétique, Paris, France

**Keywords:** primary aldosteronism, aldosterone producing adenoma, somatic mutations, potassium channels, calcium channels, ATPase, wnt/β-catenin pathway, shh signaling pathway

## Abstract

Primary aldosteronism (PA) is the most common form of secondary hypertension with an estimated prevalence of ~10% in referred patients. PA occurs as a result of a dysregulation of the normal mechanisms controlling adrenal aldosterone production. It is characterized by hypertension with low plasma renin and elevated aldosterone and often associated with hypokalemia. The two major causes of PA are unilateral aldosterone producing adenoma (APA) and bilateral adrenal hyperplasia, accounting together for ~95% of cases. In addition to the well-characterized effect of excess mineralocorticoids on blood pressure, high levels of aldosterone also have cardiovascular, renal, and metabolic consequences. Hence, long-term consequences of PA include increased risk of coronary artery disease, myocardial infarction, heart failure, and atrial fibrillation. Despite recent progress in the management of patients with PA, critical issues related to diagnosis, subtype differentiation, and treatment of non-surgically correctable forms still persist. A better understanding of the pathogenic mechanisms of the disease should lead to the identification of more reliable diagnostic and prognostic biomarkers for a more sensitive and specific screening and new therapeutic options. In this review, we will summarize our current knowledge on the molecular and cellular mechanisms of APA development. On one hand, we will discuss how various animal models have improved our understanding of the pathophysiology of excess aldosterone production. On the other hand, we will summarize the major advances made during the last few years in the genetics of APA due to transcriptomic studies and whole exome sequencing. The identification of recurrent and somatic mutations in genes coding for ion channels (*KCNJ5* and *CACNA1D*) and ATPases (*ATP1A1* and *ATP2B3*) allowed highlighting the central role of calcium signaling in autonomous aldosterone production by the adrenal.

## Background

**Figure 1:**
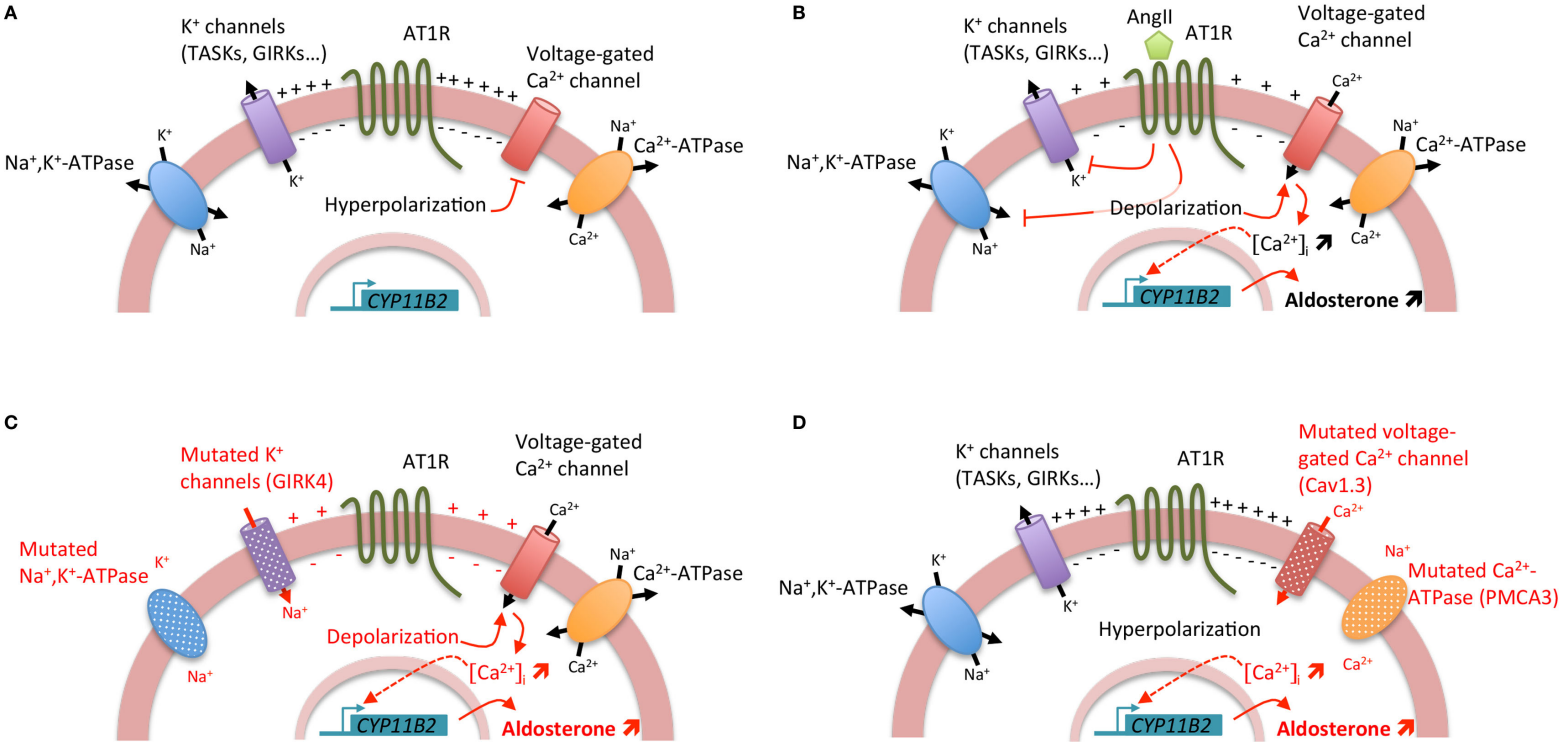
**Regulation of aldosterone biosynthesis in normal and pathological conditions**. **(A)** Under resting conditions, zona glomerulosa cells exhibit a strongly negative membrane potential (−80 mV) due to the expression of a large number of potassium channels. **(B)** Stimulation of aldosterone biosynthesis by AngII. The binding of AngII to the AngII type I receptor (AT1R) induces a cascade of events leading to the zona glomerulosa cell depolarization and the increase of intracellular Ca^2+^ concentration. The inhibition of potassium channels and Na^+^, K^+^-ATPase by AngII results in zona glomerulosa cell depolarization, opening of voltage-gated Ca^2+^ channels, and increase of intracellular Ca^2+^ concentration. Furthermore, activation of AT1R leads also to the increase of inositol triphosphate formation and consequently to the release of Ca^2+^ from the endoplasmic reticulum. Activation of the calcium signaling pathway triggers a phosphorylation cascade, involving calmodulin and calmodulin-dependent kinase I/IV, leading to the activation of specific transcription factors that bind to the promoter region and positively regulate the transcription of *CYP11B2* leading to an increase in aldosterone biosynthesis. **(C)** Genetic alterations in *KCNJ5* (coding for the potassium channel GIRK4) and *ATP1A1* (encoding the α1 subunit of the Na^+^, K^+^-ATPase) genes lead to cell membrane depolarization triggering opening of voltage-gated Ca^2+^ channels and consequently positive regulation of *CYP11B2*. **(D)** Genetic alterations in *ATP2B3* (coding for the plasma membrane Ca^2+^ ATPase, PMCA3) and *CACNA1D* (encoding the Cav1.3 subunit of the L-type voltage-gated Ca^2+^ channel) genes lead directly to the increase of intracellular Ca^2+^ concentration by affecting calcium recycling and influx, resulting in positive regulation of CYP11B2.

Aldosterone is synthesized from cholesterol by a series of specific enzymatic reactions in the zona glomerulosa of the adrenal cortex; the final steps are catalyzed by the aldosterone synthase (encoded by *CYP11B2*). Aldosterone production from the adrenal cortex is tightly controlled to maintain electrolyte and fluid homeostasis; the two principal secretagogues are the renin/angiotensin system and the extracellular concentration of potassium (K^+^). The stimulation by angiotensin II or K^+^ results in depolarization of the zona glomerulosa cell membrane and opening of voltage-gated calcium (Ca^2+^) channels, leading to an increase of intracellular Ca^2+^ concentration. Angiotensin II, by its binding to the angiotensin II type I receptor (AT1R), also acts by increasing inositol triphosphate formation leading to the release of Ca^2+^ from the endoplasmic reticulum. Activation of the calcium signaling pathway triggers a phosphorylation cascade, involving calmodulin and calmodulin-dependent kinase I/IV, leading to the activation of specific transcription factors (NURR1, NGF1B, CREB) that bind to the promoter region and positively regulate the transcription of *CYP11B2* leading to an increase in aldosterone biosynthesis (Figure 1) (1). Hence, the activation of hormone synthesis is Ca^2+^ dependent, and the regulatory mechanism involves Ca^2+^ mediated processes.

Deregulation of the mechanisms regulating aldosterone biosynthesis results in primary aldosteronism (PA), the most common form of secondary hypertension with an estimated prevalence of about 10% in referred patients and 4% in primary care (2) and as high as 20% in patients with resistant hypertension (3). PA is characterized by hypertension with elevated plasma aldosterone and low plasma renin levels, and often associated with hypokalemia. The two major causes of PA are unilateral aldosterone producing adenoma (APA) and bilateral adrenal hyperplasia (BAH), accounting together for ~95% of cases. The early detection of PA has an important impact on clinical outcome and survival given the major cardiovascular adverse effect of aldosterone excess, which is independent of blood pressure (BP). Patients with PA have been reported to exhibit more severe left ventricular hypertrophy and diastolic dysfunction than patients with essential hypertension and a high prevalence of myocardial infarction, stroke, and atrial fibrillation (4, 5). Despite the publications in 2008 of guidelines for the management of PA, there remain a few critical issues related to diagnosis, subtype differentiation, and treatment of non-surgically correctable forms (6). A better understanding of the pathogenic mechanisms of the disease should lead to the identification of more reliable diagnostic and prognostic biomarkers for a more sensitive and specific screening and new therapeutic options.

During the last few years, major advances have been made in understanding the genetic basis of APA, with the identification of mutations in genes coding for ion channels [*KCNJ5*, coding for the G protein-activated inward rectifier potassium channel 4 (GIRK4) (7) and *CACNA1D*, encoding the Cav1.3 channel (calcium channel, voltage-dependent, L type, alpha 1d subunit) (8, 9)] and ATPases [*ATP1A1*, coding for the α1 subunit of the Na^+^/K^+^-ATPase (9, 10) and *ATP2B3* encoding the plasma membrane Ca^2+^-ATPase, type 3 (10)] in more than 50% of APA. Interestingly, all these mutations lead to the activation of calcium signaling, the major trigger for aldosterone production (Figure 1). However, if the role of these mutations in regulating aldosterone production has been clearly established, their implication in proliferation and APA formation are still matter of debate (11).

In this review, we will summarize our current knowledge on the molecular and cellular mechanism of APA development. We will discuss how various animal models have improved our understanding of the pathophysiology of excess aldosterone production. We will also summarize the major advances made during the last few years in the comprehension of the genetic basis of APA formation using omics approaches, highlighting the major role of the ionic equilibrium and regulation of cell membrane potential in autonomous aldosterone overproduction.

## Ionic Equilibrium and Membrane Potential Regulation

The regulation of cell membrane potential of the zona glomerulosa is crucial to maintain the cell in a hyperpolarized state in the absence of a secretagogue stimulus. The zona glomerulosa cell membrane is selectively permeable to K^+^, giving it the characteristics of a K^+^ electrode over a wide range of extracellular K^+^ concentrations, due to the expression of a large number of potassium channels. However, their major role in the development of APA was highlighted only recently by the identification of somatic and germline mutations in genes coding for proteins involved in ionic equilibrium and membrane potential regulation but also by the establishment and analysis of mouse models in which the expression of specific potassium channels was invalidated.

### Alteration of ionic equilibrium in APA

In 2011, by a whole exome sequencing approach, few recurrent somatic *KCNJ5* mutations were identified (7). These mutations (p.Gly151Arg and p.Leu168Arg) are located near or within the selectivity filter of the channel GIRK4. Additional mutations in or surrounding the selectivity filter have been identified, including p.Gly151Glu, p.Thr158Ala, p.Glu141Gln, p.Ile157Ser, delIle157, InsThr149 (12–16). All these mutations result in a significant decrease in K^+^ selectivity and greater influx of Na^+^ into the cell, resulting in chronic cell depolarization followed by opening of voltage-dependent calcium channels and activation of calcium signaling and aldosterone production (11, 17). Germline *KCNJ5* mutations were also identified as the causative event of Familial hyperaldosteronism type III (FH-III). FH-III was first described in 2008 in a father and two daughters with early-onset severe arterial hypertension resistant to medical treatment and hypokalemia (18). To control BP, a bilateral adrenalectomy was required for all three individuals; histology revealed massive hyperplasia of the adrenal cortex (18). Further exome sequencing performed on APA allowed the identification of other somatic mutations in genes coding for ATPases, namely *ATP1A1* (9, 10) and *ATP2B3* (10) and the Cav1.3 calcium channel, *CACNA1D* (8, 9). Whereas mutations in *KCNJ5* and *ATP1A1* affect adrenal zona glomerulosa cell membrane potential and intracellular ionic homeostasis, with chronic depolarization leading to opening of voltage-dependent calcium channels and activation of calcium signaling and aldosterone production (7, 9–11), mutations in *ATP2B3* and *CACNA1D* modify directly intracellular calcium equilibrium, also leading to an activation of calcium signaling and aldosterone production (Figure 1) (8–10).

### Prevalence of somatic mutations and genotype/phenotype correlations

The prevalence of somatic mutations in APA has been extensively investigated in many studies (9, 10, 14, 19–22). *KCNJ5* mutations are the most frequent genetic abnormalities reported in APA with a prevalence of ~40% in Caucasian population, and as high as 70% in series from Japan (21, 23). The mutations affecting *ATP1A1* and *ATP2B3* genes are less frequent with a reported prevalence of 5.3 and 1.7, respectively (9, 10, 20). Mutations in the *CACNA1D* gene are the second most frequent genetic alterations observed in APA with a prevalence comprised between 5 and 9.3% (8, 9, 20). Interestingly, whereas mutations in *KCNJ5*, *ATP1A1*, and *ATP2B3* are located in specific “hot spots,” a large number of mutations were reported in different exons of the *CACNA1D* gene, affecting more frequently segment M4 and M6 of the protein, implying the necessity of a large genotyping of *CACNA1D* in APA. Different studies established correlations between clinical and biological parameters and the mutational status of the tumor (10, 19, 20). Hence, patients with *KCNJ5* mutations were more frequently female and diagnosed younger than patients harboring *CACNA1D* mutations and non-carriers (20); and *CACNA1D* mutations associated with smaller adenoma size (9, 20). Some studies reported also association between the mutational status and cellular composition of the adenoma. APA harboring *KCNJ5* mutations would be composed essentially of zona fasciculata-like cells whereas those carrying *CACNA1D* mutations of a majority of zona glomerulosa-like cells (9), although this association was not replicated in all series (20). The exploration of the relationship between adrenal cortex remodeling and *KCNJ5* mutations revealed the absence of association between the *KCNJ5* mutational status and the nodulation score in the peritumoral tissue, the vascularization and the presence of zona glomerulosa hyperplasia in the peritumoral cortex, suggesting that *KCNJ5* mutations are not likely to be responsible for a specific microenvironment propitious to promote adrenal cortex remodeling and APA formation (24).

### Lessons from potassium channel knock-out mouse models

Though the role of all these mutations in abnormal aldosterone secretion has been clearly established, their impact in adenoma formation still remains unclear. Indeed, whereas in HAC15 cells, the overexpression of GIRK4 carrying the p.Thr158Ala mutation was responsible for a significant increase in aldosterone production, it induced, in parallel, a decrease in cell proliferation, independently of intracellular Ca^2+^ concentration (11). Likewise, the overexpression of p.Glu151Arg or p.Glu151Gln in HEK293T cells resulted in rapid Na^+^-dependent lethality (15). More extensively, a still open question is to know whether a modification in the ionic equilibrium and the regulation of the cell membrane potential are also able to promote adenoma formation. Response elements came some years ago with the investigation of mouse models in which TASK1 and/or TASK3 potassium channels were invalidated to determine the contributions of TASK channels to background K^+^ currents in adrenal zona glomerulosa cells and test their role in the control of aldosterone production (25, 26). TASK1 and TASK3 are two-pore domain K^+^ channels (K2P) that contribute largely to the very high background conductance of zona glomerulosa cells, making of zona glomerulosa cells highly sensitive sensor for plasma K^+^ concentration. They clamp the cell membrane to hyperpolarized voltages, restraining the production of aldosterone in absence of stimulus. In mouse adrenal cortex, whereas TASK1 expression is found throughout the zona glomerulosa and fasciculata, TASK3 expression is restricted to zona glomerulosa (25). Deletions of task1 and task3, respectively, lead to the development of hyperaldosteronism or low-renin hypertension (25–28) In task1^−/−^ mice, hyperaldosteronism was due to aberrant functional zonation of the adrenal cortex, with intense *cyp11b2* expression being localized in zona fasciculata instead of the zona glomerulosa. Interestingly, young task1^−/−^ mice exhibited PA both in males and females; after puberty, this phenotype was only observed in females. Hyperaldosteronism was modulated by sexual hormones, being corrected by testosterone administration in task1^−/−^ females and triggered by castration in males (26), suggesting that after puberty other factors, including task3 potassium channels, could substitute for the absence of task1 and promote compensatory mechanisms in male task1^−/−^ mice (26). Deletion of task3 in mice leads to low-renin salt-sensitive hypertension, with suppressed plasma renin and aldosterone secretion that is not suppressible by increasing salt intake (27). Primary cultures of adrenocortical cells of these mice were strongly depolarized when compared with wild-type mice, and in fresh adrenal slices, calcium signaling was abnormal in zona glomerulosa cells (28). Finally, deletion of both task1 and task3 results in a marked depolarization of the zona glomerulosa cell membrane potential and a mild hyperaldosteronism with plasma aldosterone levels stimulated by a low-sodium diet but not suppressed by a high-sodium diet and partially responsive to AngII blockade (25). Interestingly, invalidation of these different potassium channels leads to hyperaldosteronism due to abnormal depolarization of the zona glomerulosa cell membrane resulting in increased intracellular Ca^2+^ concentration and stimulation of aldosterone biosynthesis; however formation of adrenal tumors has never been observed in these models indicating that other mechanisms are required to promote increased cell proliferation in APA. Although the invalidation of task1 and task3 in mice resulted in hyperaldosteronism or low-renin hypertension, to date no mutation in *KCNK3* or *KCNK9* genes has been reported in APA. However a reduced expression of TASK2, encoded by *KCNK5*, has been recently described in APA compared with normal adrenal (29), and the expression in H295R cells of a TASK2 dominant-negative mutant resulted in increased aldosterone production and *CYP11B2* and *StAR* expression. Comparison of gene expression profiles of adrenal glands of task1^−/−^ female and male mice allowed the identification of a cluster of genes closely associated with hyperaldosteronism (30), among them dickkopf3 (Dkk3), a member of the dickkopf family of Wnt signaling modulators. Inactivation of dkk3 in task1^−/−^ mice resulted in the extension of the phenotype of hyperaldosteronism to male animals, without inducing abnormal zonation of the adrenal cortex (30). Interestingly, the expression of Dkk3 was found to be frequently downregulated in almost any cancer entity and emerged as a potential key player in tumor suppression (31). These results suggest that the Wnt/β-catenin pathway could play a role in the development of APA.

## Activation of Sonic HedgeHog and Wnt/β-Catenin Pathway: Common Features of APA

The role of specific mutations of channels and ATPases in affecting aldosterone biosynthesis is now clearly established, whereas the question of the mechanism responsible for abnormal proliferation leading to adenoma formation is still open. In 2011, Lifton suggested that *KCNJ5* mutations could be responsible for both autonomous aldosterone production and abnormal cell proliferation (7); however it has been rapidly shown that cells expressing mutated *KCNJ5* channels were less proliferative (11), raising the questions as to the events leading to abnormal cell proliferation and adenoma formation? Two specific pathways are known to play a crucial role in adrenal development: the Sonic HedgeHog and the Wnt/β-catenin pathways.

### Wnt signaling pathway in proliferation and/or aldosterone biosynthesis

#### The Canonical and Non-Canonical Wnt/β-Catenin Pathway

Wnt signaling has been shown to be a key signaling pathway in both normal adrenal development and tumorigenesis. The “canonical Wnt signaling pathway” acts through the regulation of the amount of the transcriptional regulator β-catenin, which controls the expression of specific genes involved in development. In the absence of Wnt, β-catenin is a part of the axin complex consisting of adenomatous polyposis coli (APC), axin, glycogen synthase kinase-3β (GSK-3β), and casein kinase-1β (CK-1β). CK-1β and GSK-3β sequentially phosphorylate β-catenin in its N-terminal part resulting in its ubiquitination and degradation by the proteasome, thus preventing β-catenin from translocation to the nucleus and activation of specific Wnt target genes. The Wnt/β-catenin activation occurs through the binding of Wnt ligand to its cell surface receptor consisting of a frizzled receptor and its co-receptor, the low-density lipoprotein receptor related protein (LRP) 6 or LRP5. Activation of the receptor leads, through an unknown mechanism, to the phosphorylation of the disheveled (Dvl) protein, which prevents GSK-3β from phosphorylating specific substrates such as axin, APC, and β-catenin. Hence, the binding of Wnt ligands to their receptor results in the inhibition of β-catenin phosphorylation, dissociation from the axin complex, accumulation in the cytoplasm and translocation to the nucleus where it serves as a transcriptional coactivator of transcription factors of the T-cell factor (TCF)/lymphocyte enhancer factor (LEF) family. TCF/LEF target genes are involved in regulating cell proliferation, stem cells maintenance, and differentiation. To increase the complexity of the system, Wnt signaling independent of β-catenin has been described as “non-canonical Wnt signaling pathway.” It implicates small GTPases/jun N-terminal kinase (JNK) and intracellular calcium signaling (32). Finally, the activation of the Wnt pathway can be antagonized by specific natural molecules including secreted Frizzled-related proteins (sFrps) and Dickkopk (Dkk) family members. SFrps and Dkk are secreted proteins acting on different components of the Wnt signaling pathway. sFrps display a high sequence homology with the Wnt binding site of Frizzled allowing sFRP proteins to directly bind to Wnts, thus functioning as Wnt antagonists for both “canonical” and “non-canonical” pathways (33), whereas Dkk members are not only able to inhibit the Wnt coreceptors LRP5 and 6 but also to bind with high affinity to the transmembrane proteins Kremen 1 and 2, which also modulate Wnt signaling (33, 34).

#### Role of Wnt/β-Catenin Pathway in Adrenal Function

The Wnt/β-catenin pathway plays an important role in embryonic development, stem cell maintenance, and differentiation in many tissues. During the two last decades, the role of Wnt/β-catenin in adrenal development has been highlighted by the exploration of different mouse models in which expression of different components of the pathway were disrupted (35–37) or constitutively activated (38). The first element indicating a role of this pathway in adrenal is the localization of some of its components (i.e., β-catenin, wnt4, dkk3, sfrp1…) specifically in the subcapsular zone and in zona glomerulosa (39, 40). The loss of Wnt4 was associated with abnormal differentiation of the definitive zone of the adrenal cortex and aberrant migration of adrenocortical cells into the developing gonad (35) and with a decrease of the number of zona glomerulosa cells which results in a decrease of aldosterone production (36). Interestingly, the expression of Wnt4 mRNA has been reported to be higher in APA than in normal adult adrenocortical cells (41). Overexpression of WNT4 in human adrenocortical cells resulted in an increase of aldosterone biosynthesis, whereas DKK3 had an inhibitory effect, suggesting that Wnt/β-catenin pathway could be also involved in glomerulosa specific functions (42).

#### Modulation of β-Catenin Expression or Activation in the Adrenal Gland

The disruption of β-catenin specifically in adrenocortical cells, through the use of a sf-1 (steroidogenic factor-1)-Cre mouse, resulted in complete adrenal aplasia or defects in maintenance of the adult cortex resulting in depletion of adrenocortical cells (37). Inversely, the constitutive activation of β-catenin in the adrenal cortex resulted in profound adrenocortical zonation defects characterized by an ectopic activation of the zona glomerulosa differentiation program and inhibition of orthotopic zona fasciculata differentiation. Interestingly, at the age of 10 months, these mice develop hyperaldosteronism (38) similarly to mice expressing a defective APC allele (43), suggesting that constitutive activation of the Wnt/β-catenin pathway could play a role in the development of APA. In human adrenal, while β-catenin expression was found in the entire cortex, its activated form was restricted to zona glomerulosa cells (44), suggesting that restriction of β-catenin activation to sub-capsular regions and in zona glomerulosa is necessary for the development of functional zonation in the human adrenal cortex. Studies in human adrenocortical cells have indicated that Wnt signaling molecules may also have multiple actions on steroidogenesis, particularly in regulating aldosterone biosynthesis (36, 45). All these results suggest that aberrant Wnt signaling may be driving the development of APA. Recently, the activation of Wnt/β-catenin has been reported in two-thirds of APA (44, 46). Whereas activating mutations of the β-catenin are found in a wide variety of human cancers including adrenocortical tumors and adrenocortical adenoma, only few mutations were reported in APA (8, 47) strongly suggesting that the activation of β-catenin was not associated with the presence of mutation (47–49). Moreover, in adrenocortical carcinoma, the activation of β-catenin was associated with a poor prognosis (49), whereas in APA it was not associated with specific tumor characteristics. Thus Wnt/β-catenin activation may play distinct roles in APA compared to adrenal cortex carcinoma, contributing to aldosterone hypersecretion rather than to autonomous cell proliferation (46). The activation of β-catenin was not only associated with an increased expression of specific target genes, i.e., *AXIN2* and *LEF1*, but also with down regulation of SFRP2, a member of the SFRP family of Wnt signaling inhibitors (46). Interestingly, s*frp2* knockout mice exhibit an increase in plasma aldosterone concentration, associated with ectopic expression of *cyp11b2* in adrenal cortex, similarly to what observed in mice expressing the constitutive active form of β-catenin in adrenal cortex (46).

### Shh signaling pathway in APA

Similarly to the Wnt/β-catenin signaling pathway, Sonic HedgeHog signaling (shh) is essential for adrenal gland development and maintenance. Shh encodes a secreted signal that belongs to the Hedgehog family. The activation of shh signaling occurs through its binding to a receptor complex formed by the twelve transmembrane domain protein patched-1 (PTCH1) and the G-protein coupled receptor Smoothened (SMO). In the presence of Shh, SMO is released from PTCH1 inhibition and activates the transcription factors GLI1, GLI2, and GLI3 (50). In rodent adult adrenals, Shh is expressed exclusively in the subcapsular region of the cortex in cells also expressing sf1, indicating their commitment to steroidogenic cells (51–53). Similarly, in human adult adrenals, the expression of SHH was found to be restricted to a few numbers of cells of the subcapsular region, where stem/progenitor cells are supposed to be localized (44, 54). Mice invalidated for Shh, specifically in Sf1 positive cells, exhibit reduced proliferation of capsular cells and a significant reduction of adrenocortex thickness and adrenal size but no modification of adrenal zonation. Moreover, the remaining adrenal cortex was able to synthetize steroids, indicating that shh is essential for expansion of the adrenal cortex but not for zonation and differentiation (55). Interestingly, the expression of Shh was found in APA as well as in the entire hyperplasic zona glomerulosa, with a similar pattern of expression than *CYP11B2* and Dab2 (44). The activation of the SHH signaling pathway in APA was confirmed by transcriptomic analysis (44). These results suggest that APA have acquired some characteristics of stem/precursor cells or, alternatively, that reexpression of fetal markers from the definitive zone in the adrenal cortex could underlie excessive proliferation and APA formation. Remarkably, the antagonism of hedgehog signaling has been shown to inhibit the proliferation of H295R cells (56) and to decrease cell viability (57). Moreover, the inhibition of shh signaling pathway results in the inhibition of wnt/β-catenin signaling (57). Interestingly, the activation of shh signaling pathway was found to be increased in adult adrenocortical carcinoma (57) as well as in non-producing adenoma (56), suggesting a role in tumor formation or development.

## Clock Genes in the Control of Aldosterone Production

Many physiological functions such as metabolism, BP, and renal function are regulated by the circadian clock (58–60). Up to 10% of the transcriptome has been estimated to be under the control of the circadian clock and a number of diseases are associated with clock gene disorders (61). The circadian timing system is organized in central and peripheral clocks. The central circadian clock is composed of specialized neurons in suprachiasmatic nuclei in the hypothalamus and is synchronized to the daily light/dark cycle through the retino-hypothalamic tract (62). The peripheral circadian clocks, found in most peripheral tissues, are synchronized to geophysical time through a wide range of master clock-dependent stimuli (62, 63). Four canonical proteins are the components of the circadian time clocks: period (Per)1–3, cryptochrome (Cry)1–2, Bmal1, and Clock. Clock and Bmal1 form a heterodimer that interacts with E-boxes to transcriptionally upregulate clock-controlled genes, which include Per and Cry (62, 64). Cry proteins act as potent transcriptional repressors that downregulate the transcription of E-box (CACGTG) enhancer-containing clock genes (including Per- and Cry-encoding genes) as well as a wide variety of clock-controlled genes (65, 66).

Different circadian mutant mice models show abnormalities in BP regulation and/or plasma aldosterone concentration. BP is decreased in *clock* knockout mice, accompanied by changes in circadian rhythms of urinary sodium and potassium excretion, and loss of the circadian rhythmicity of plasma aldosterone (67). A mouse model carrying a conditional allele of the circadian clock gene Bmal1 and expressing Cre recombinase under the endogenous Renin promoter (Bmal1lox/lox/Ren1dCre) loose the BMAL1 protein expression in the renin-secreting granular cells of the juxtaglomerular apparatus. These mice exhibit decreased BP, increased urine volume, changes in the circadian rhythm of urinary sodium excretion, and significantly reduced plasma aldosterone (63). Mice lacking the core clock components Cry1 and Cry2 (referred as Cry-null mice) show disrupted rhythmic behavior, physiology, and metabolism (68, 69). Interestingly, Cry-null mice exhibit salt-sensitive hypertension due to increased aldosterone production by the adrenal gland (70). Investigation of steroidogenic alterations in Cry-null mice showed chronic overexpression of *Hsd3b6* mRNA and chronically enhanced 3β-hydroxysteroid dehydrogenase activity in adrenal cortex. *Hsd3b6* encodes a dehydrogenase-isomerase specifically expressed in zona glomerulosa, which catalyzes the conversion of pregnenolone into progesterone, an enzymatic reaction required for aldosterone biosynthesis. The inactivation of Cry genes leads to chronically enhanced mineralocorticoid production, which, in turn, renders BP salt sensitive (70). On the other hand, it has been previously shown that Per1 and Cry2 modulate opposing actions on Per1 target gene expression in some tissues (71). Remarkably, Per1 knockout mice exhibit lower BP when compared to wild-type mice (60). To verify the hypothesis that Per1 plays a role in the regulation of aldosterone levels, Richards et al. have performed RNA silencing and pharmacological blockade of Per1 nuclear entry in the NCI-H295R human adrenal cell line, demonstrating that *Hsd3b6* expression is decreased after Per1 knockdown *in vitro* (72). In addition, they have demonstrated that Per1 heterozygous mice exhibited lower plasma aldosterone levels and reduced *Hsd3b6* mRNA expression *in vivo*, with a significant blunted circadian expression of this gene (72). In the human adrenal, two 3β-hydroxysteroid dehydrogenase isoform are expressed, namely *HSD3B1* and *HSD3B2* (70). In the adrenal cortex, expression of *HSD3B1* is specific to the zona glomerulosa (70), suggesting its potential involvement in adrenal zona glomerulosa pathophysiology. Both HSD3B1 and HSD3B2 are found to be express in APA; and whereas HSD3B2 expression was higher than that of HSD3B1 in APA, only the level of HSD3B1 expression was correlated with plasmatic aldosterone concentration and CYP11B2 expression in APA, suggesting that HSD3B1 may contribute to autonomous aldosterone production in APA (73).

## Conclusion

Despite major advances performed these last years in our understanding of the pathophysiology of APA development, the natural history of APA formation is still a matter of debate. Our current knowledge is not enough advanced to explain the mechanisms involved in APA formation. However, the identification, in about 50% of APA, of recurrent somatic mutations in genes coding for ionic channels and ATPases has elucidated the mechanism responsible for the autonomous aldosterone production. On the other hand, activation of the Wnt/β-catenin pathway or reexpression of stem/precursor cell markers, i.e., shh, could explain the abnormal proliferation leading to the formation of an adenoma. It is possible that APA formation is the result of the combination of two events: (1) the activation of signaling pathways such as wnt/β-catenin or shh pathways driving abnormal cell proliferation and creating a favorable environment for (2) the occurrence of recurrent somatic mutations responsible for autonomous aldosterone production.

Although it was suggested that genetic alterations leading to abnormal calcium signaling are sufficient for both abnormal proliferation and inappropriate aldosterone production in APA, there are some evidences suggesting that mutations in potassium and calcium channel and ATPases may not be sufficient for promoting cell proliferation and tumor formation. It could be speculated that some groups of cells start to abnormally proliferate creating a propitious environment for the emergence of specific mutations affecting ionic channels and ATPases leading to increased aldosterone production. Further mechanistic insight may come from specific mouse models developing a phenotype of hyperaldosteronism in the context of an APA.

## Conflict of Interest Statement

The authors declare that the research was conducted in the absence of any commercial or financial relationships that could be construed as a potential conflict of interest.
